# Supplementary figures and images for: Critical Differential Expression Assessment for Individual Bulk RNA-Seq Projects
Source: bioRxiv. 2024 Feb 12:2024.02.10.579728. Preprint. [Version 1] doi: 10.1101/2024.02.10.579728 (PMC10888899; doi:10.1101/2024.02.10.579728)

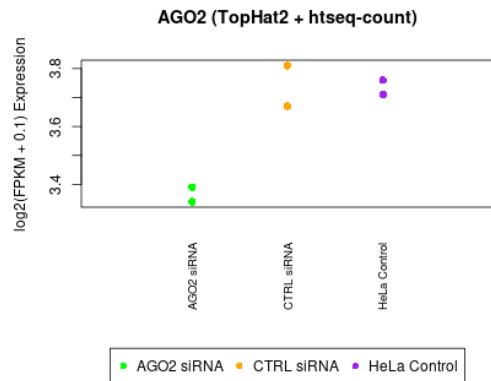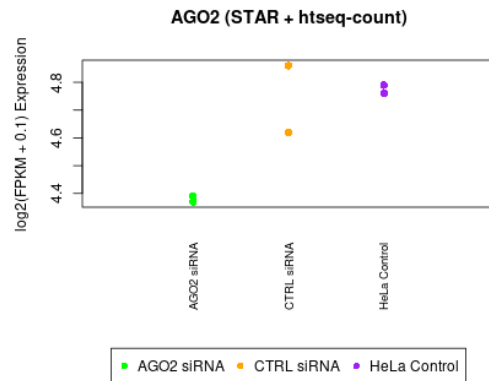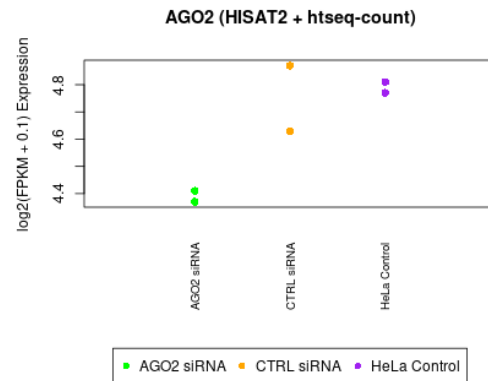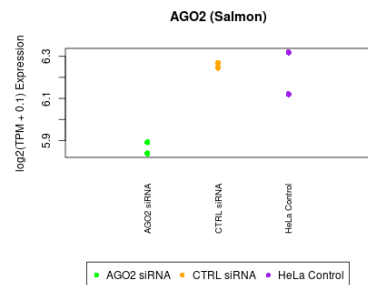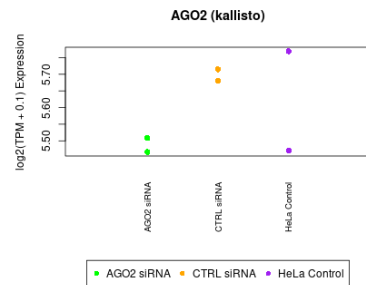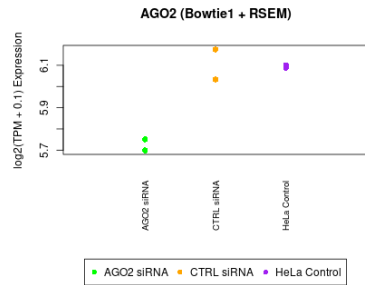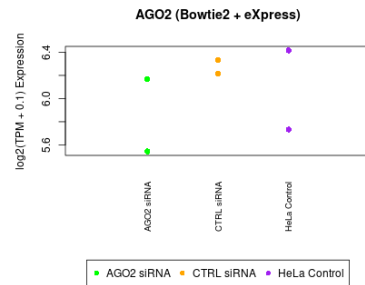

Supplement: Supplement 2 [file media-2.pdf]

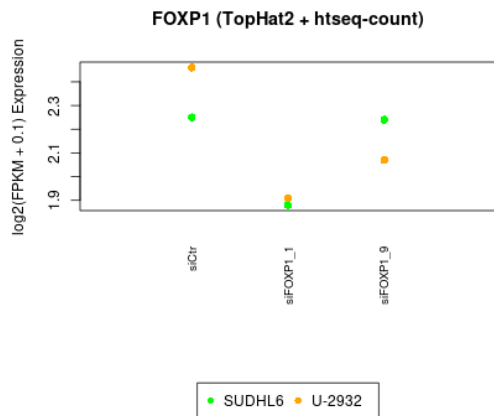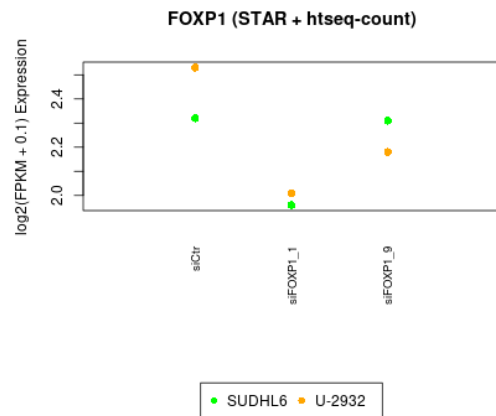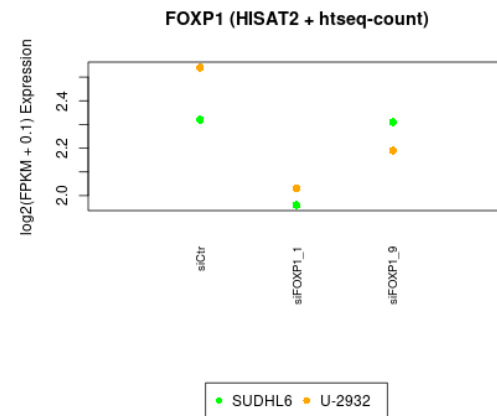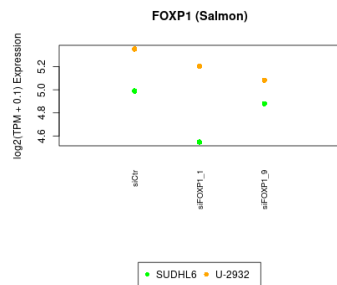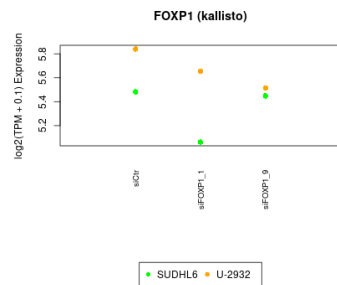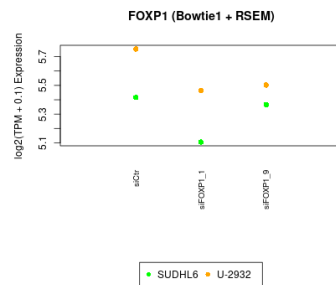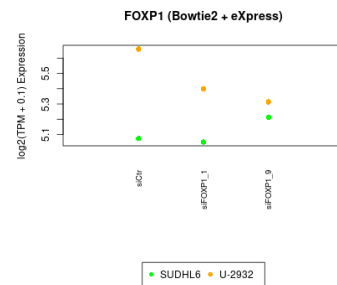

Supplement: Supplement 3 [file media-3.pdf]

**A.**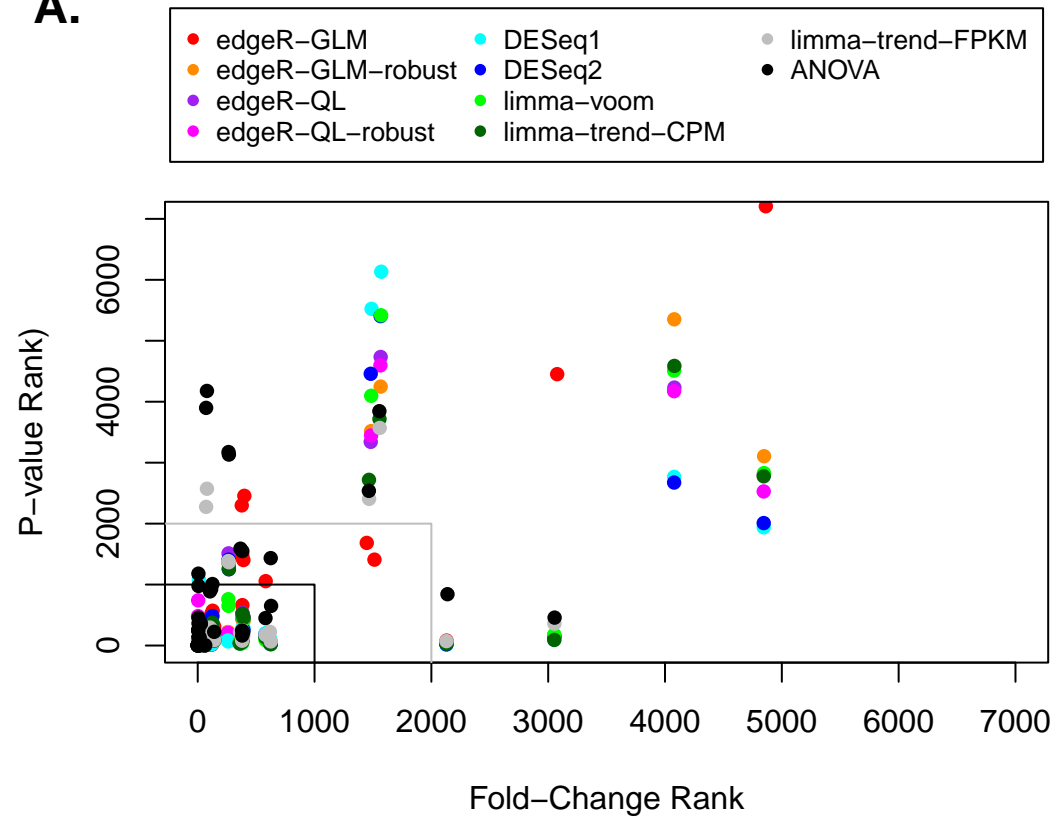**B.**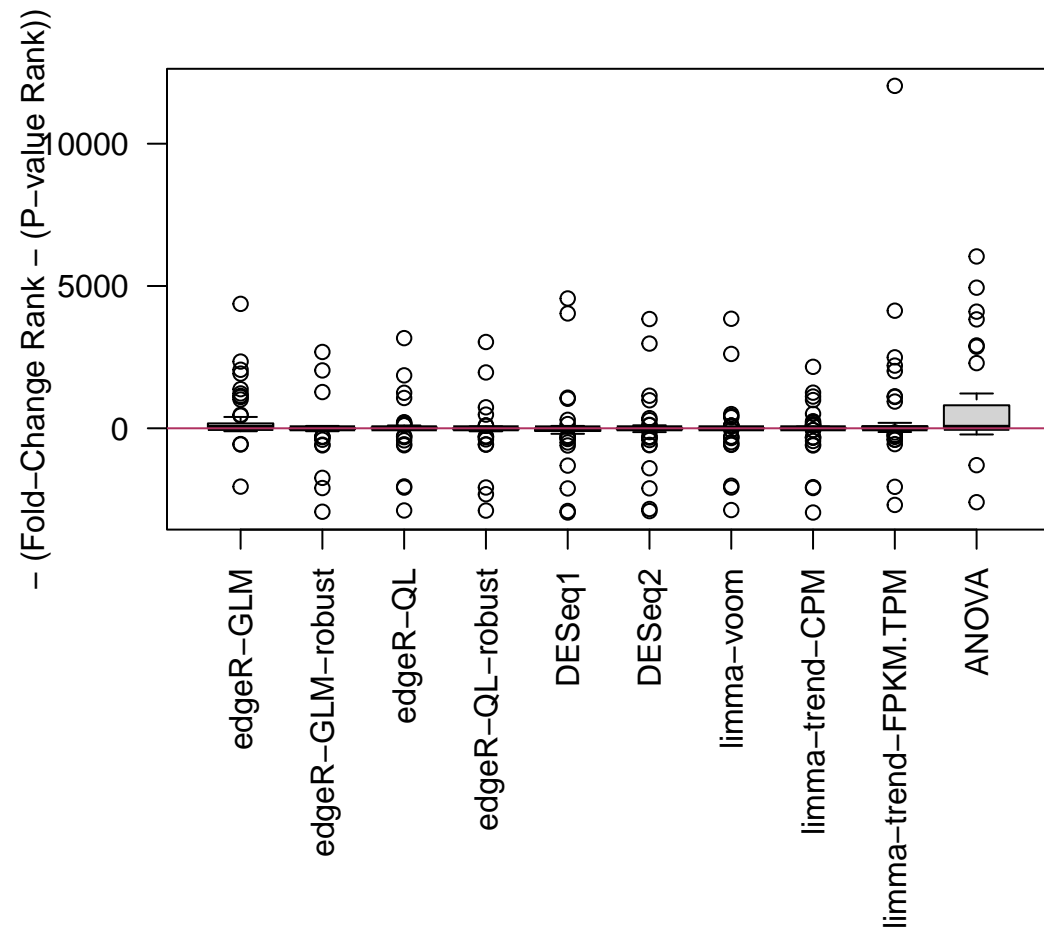

Supplement: Supplement 4 [file media-4.pdf]

**A.**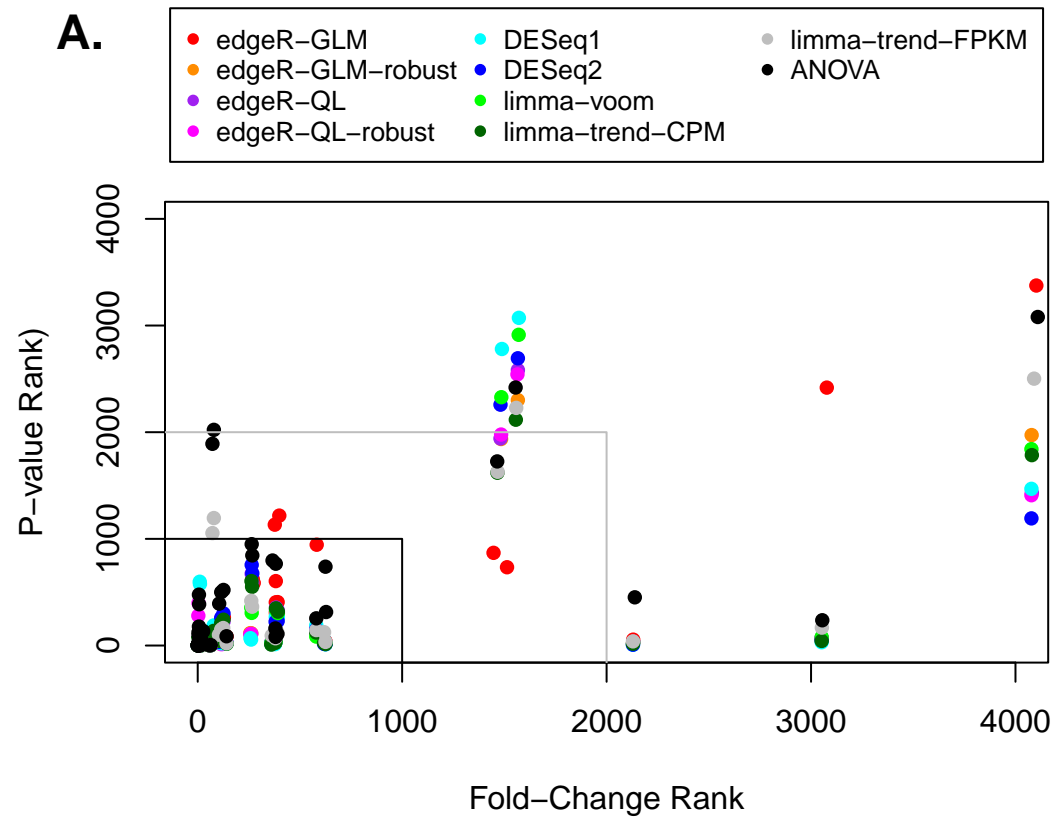**B.**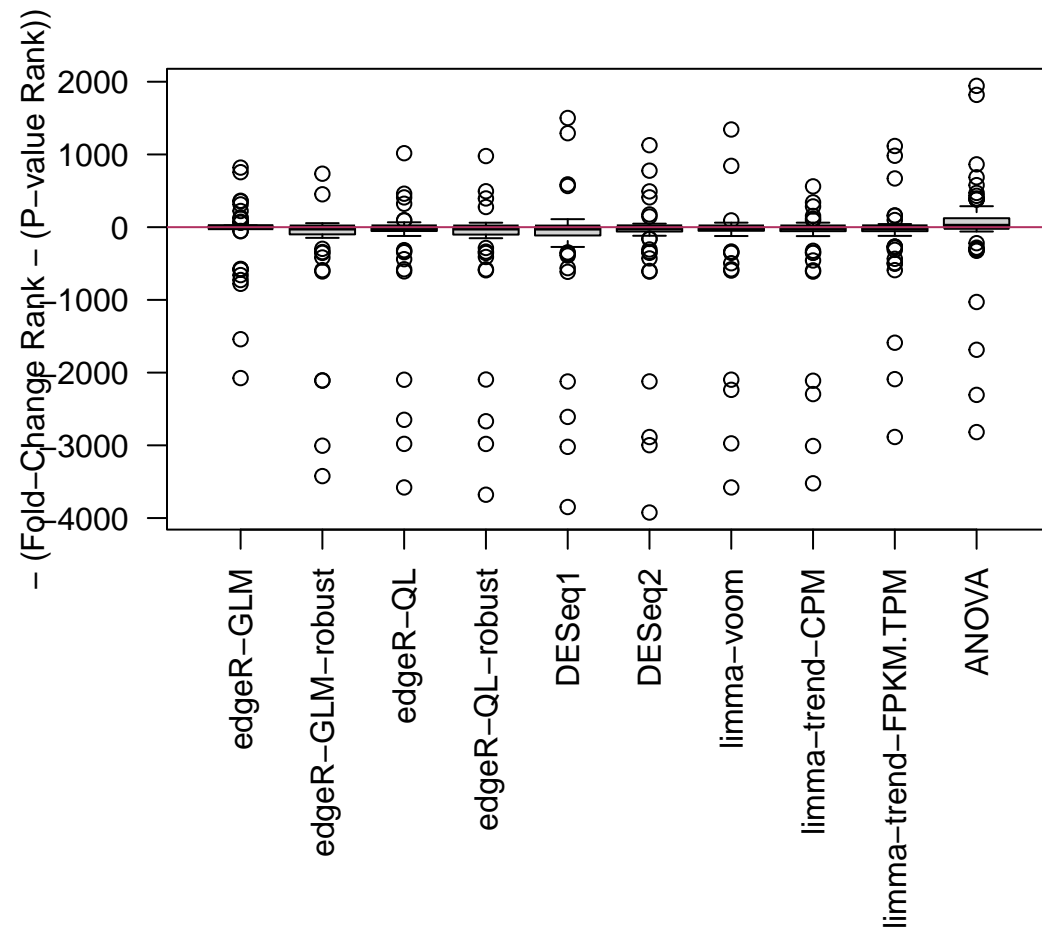

Supplement: Supplement 5 [file media-5.pdf]

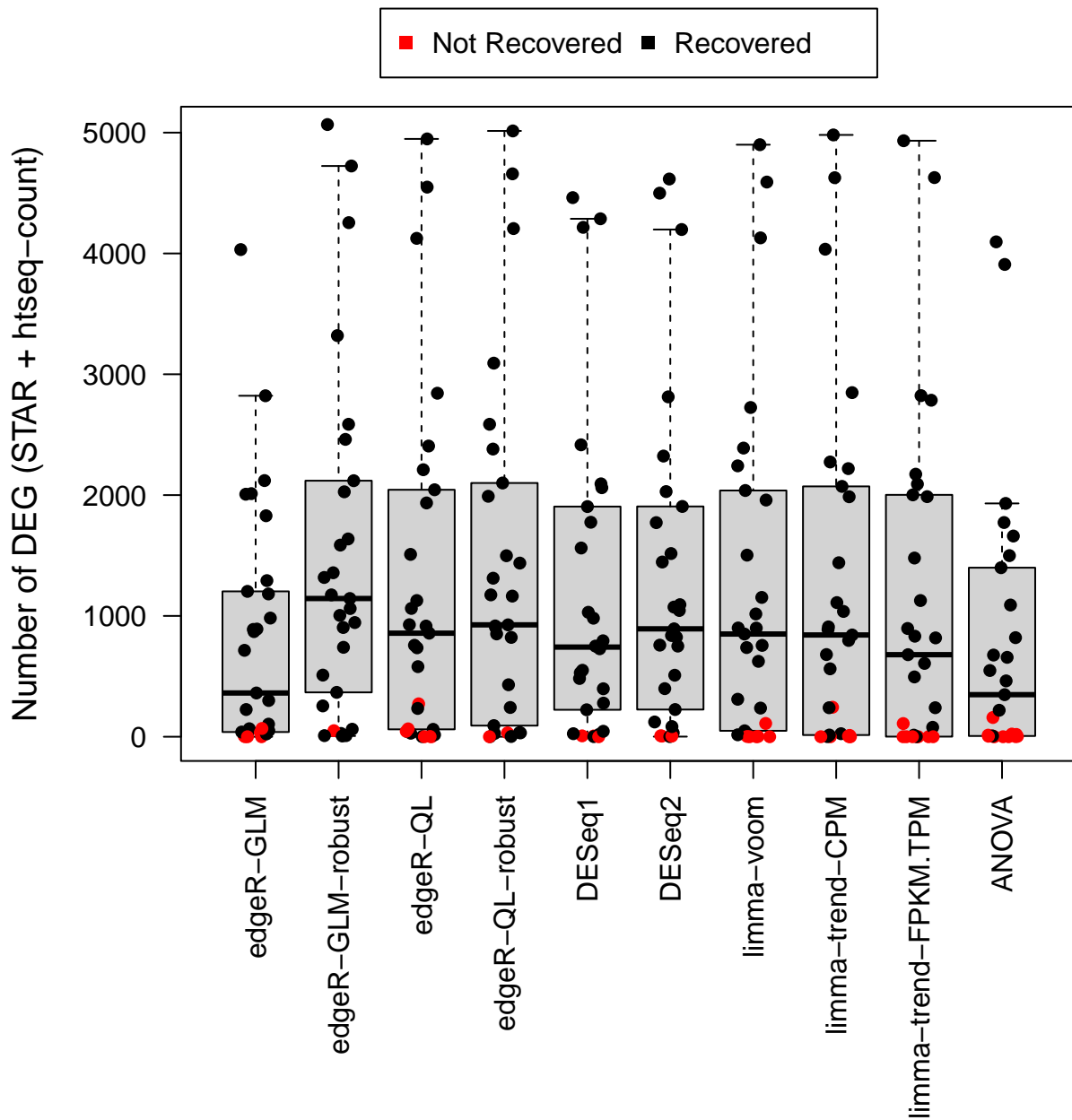

Supplement: Supplement 10 [file media-10.pdf]

Effect of Robust Dispersion for GLM

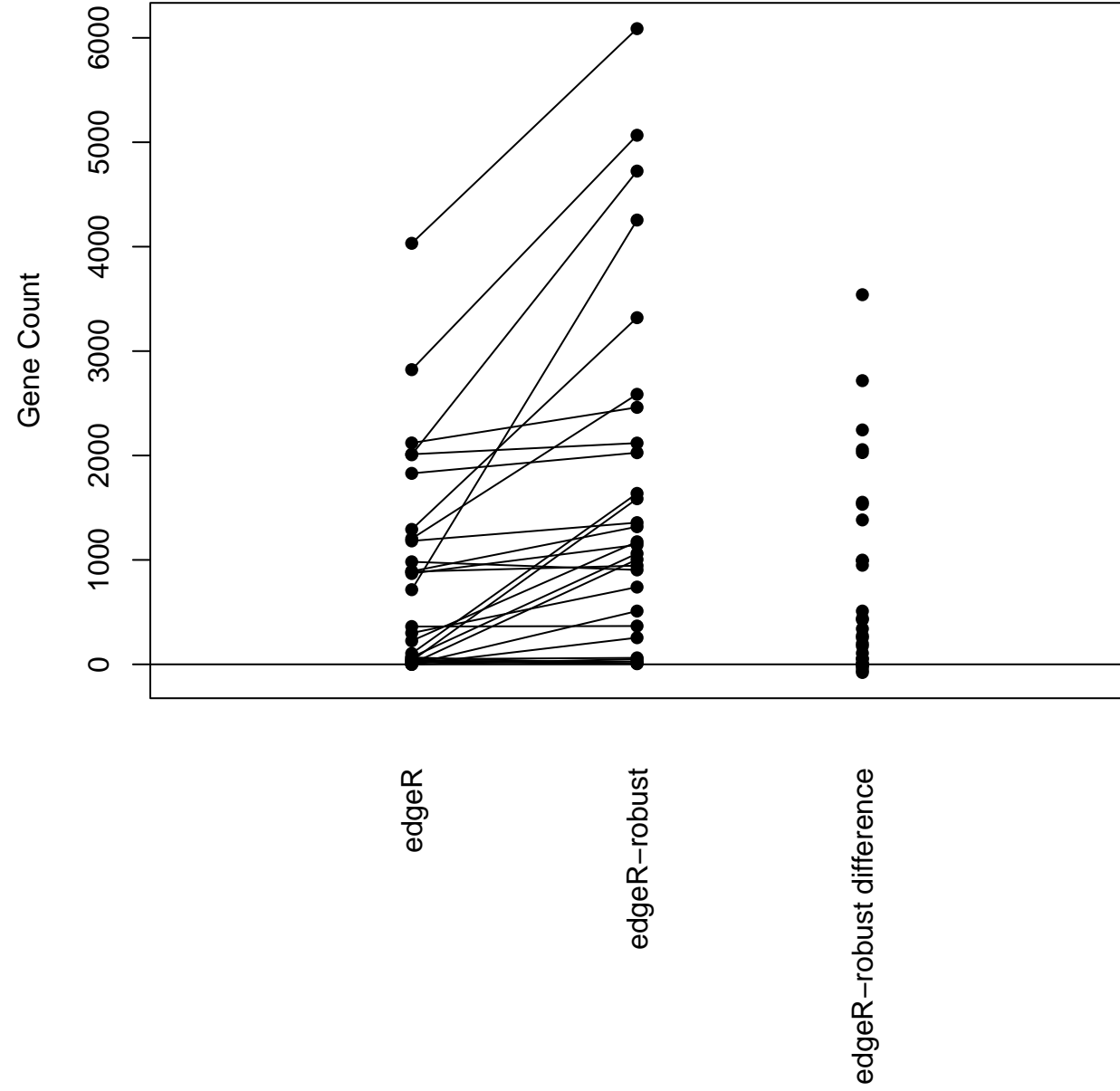

Effect of Robust Dispersion for QL

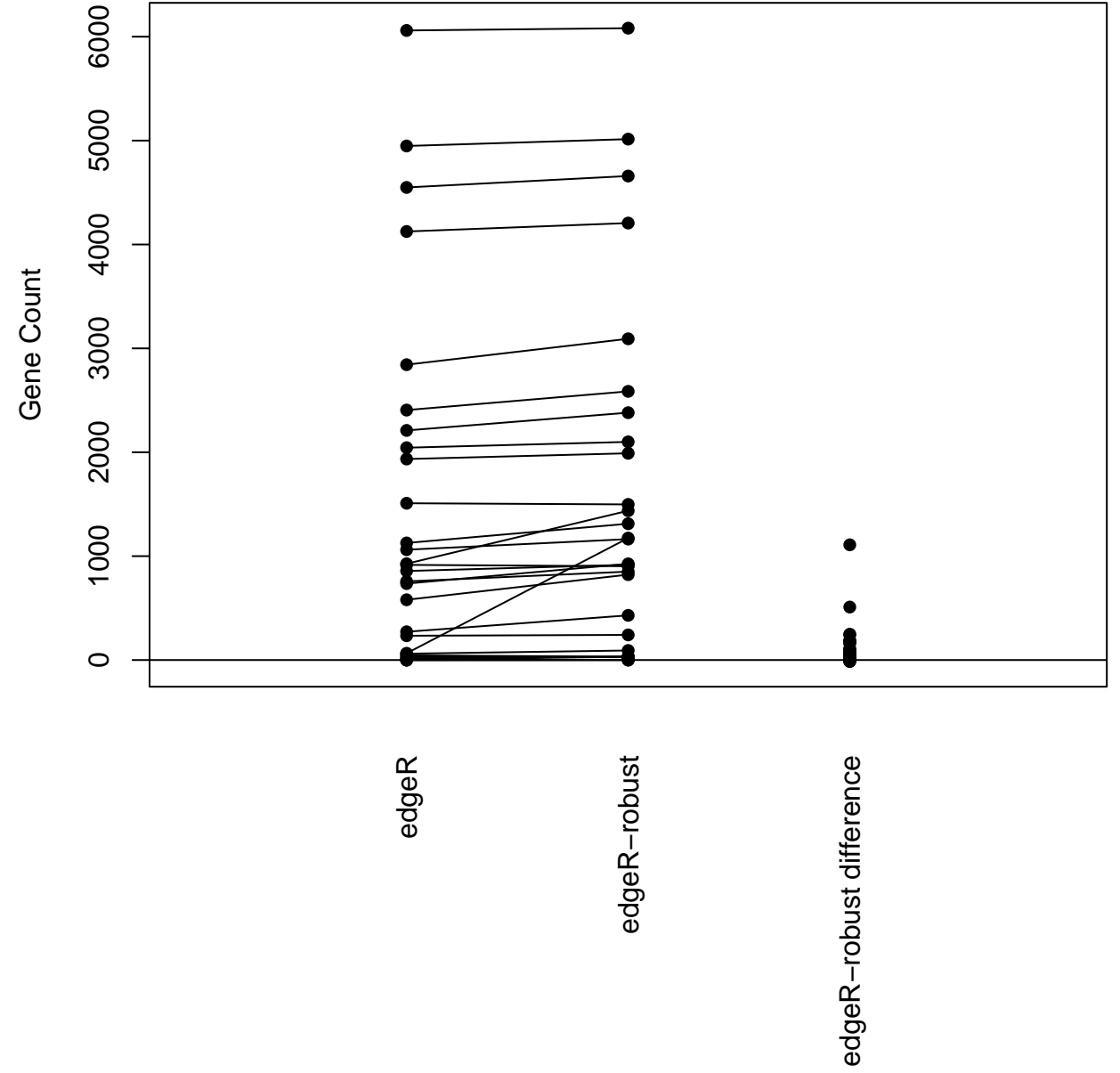

Supplement: Supplement 11 [file media-11.pdf]

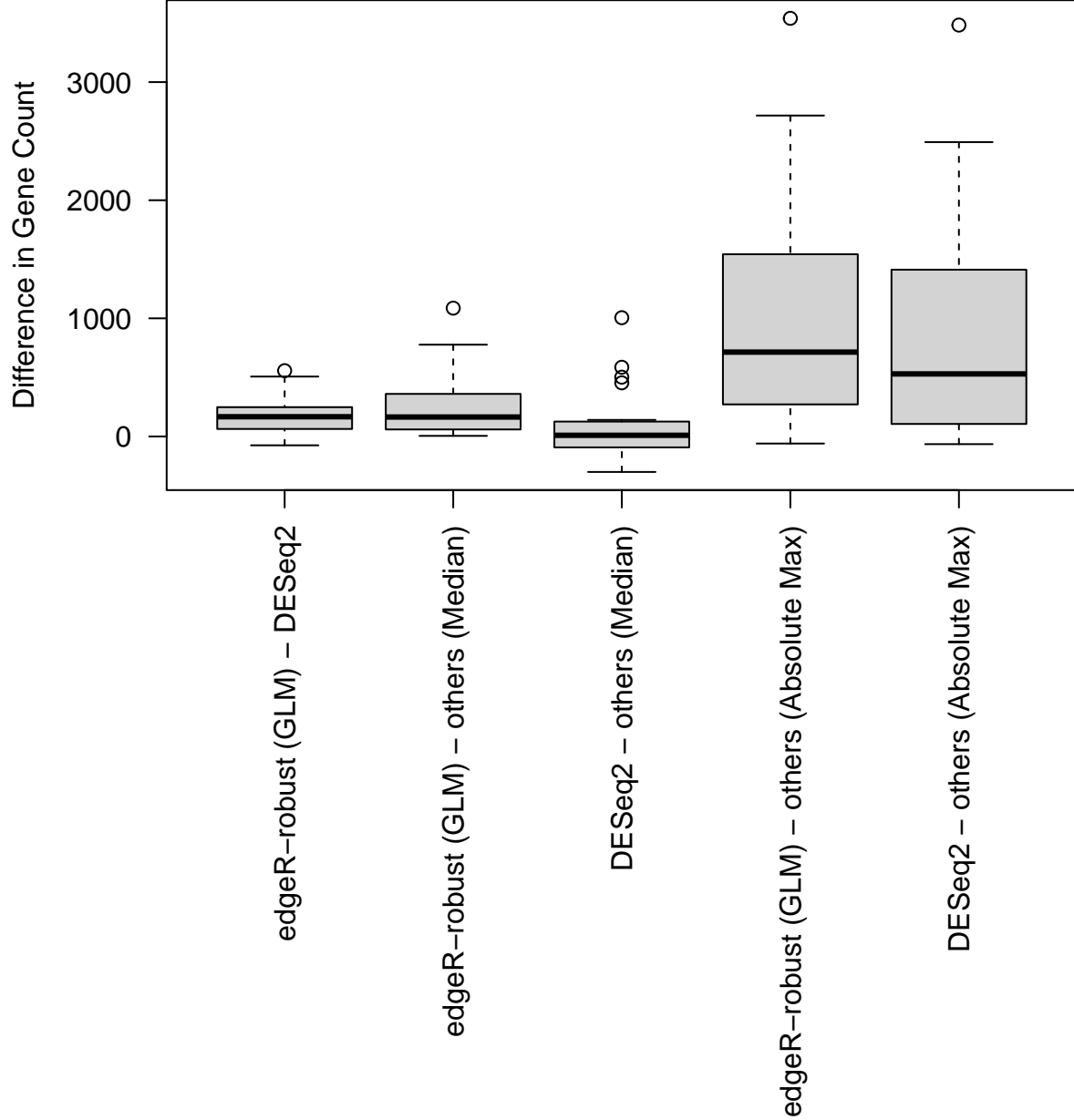

Supplement: Supplement 12 [file media-12.pdf]

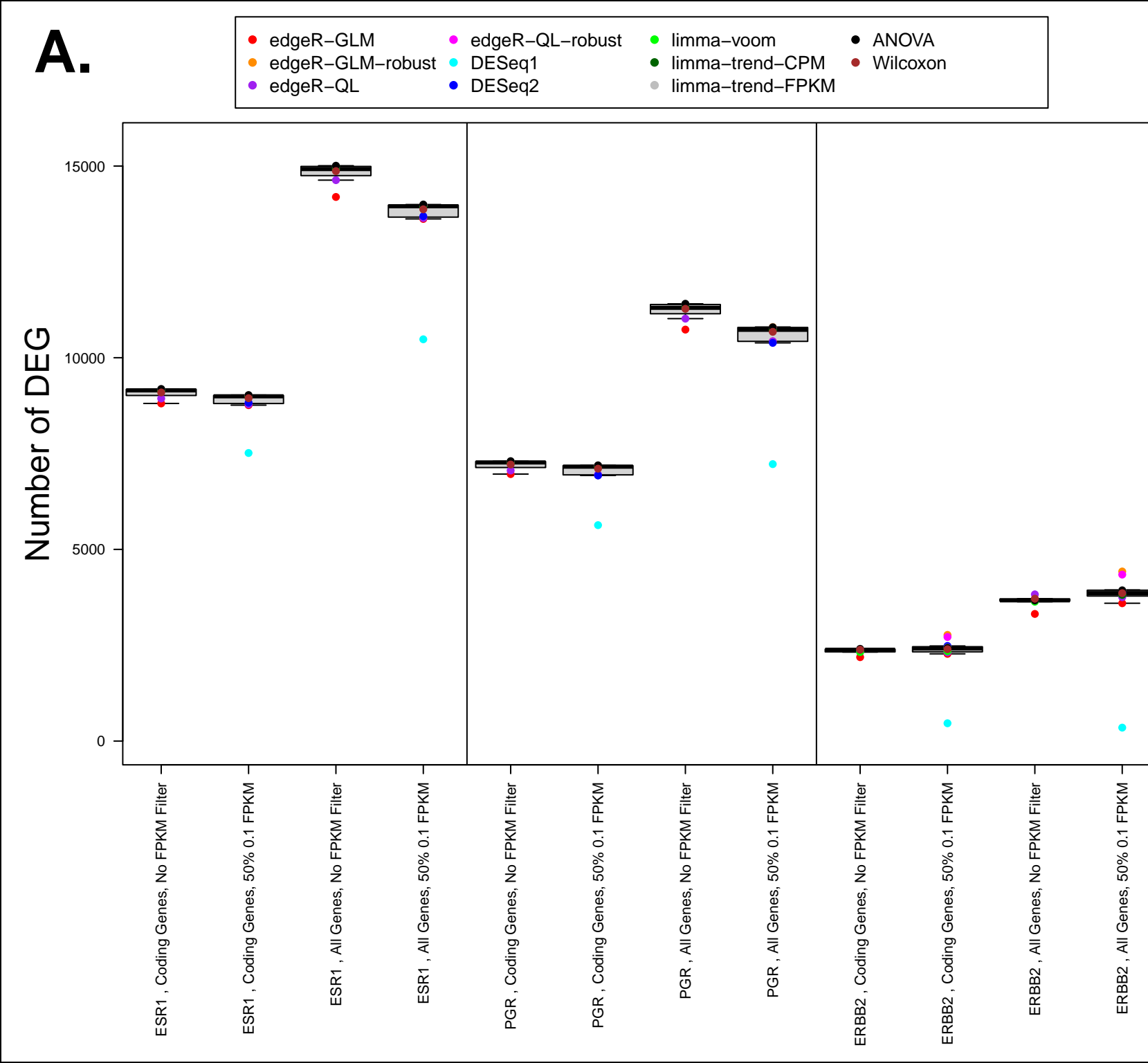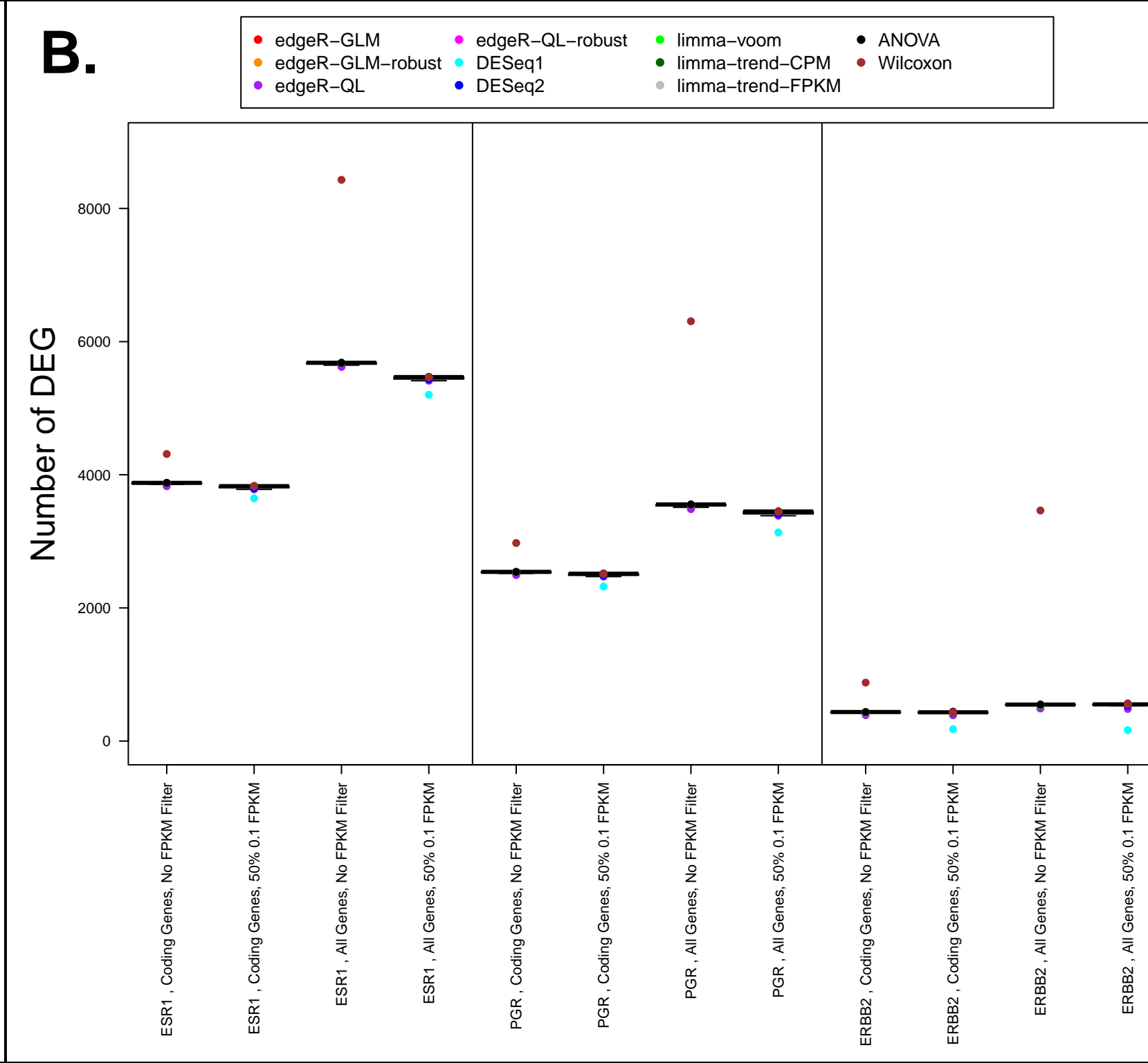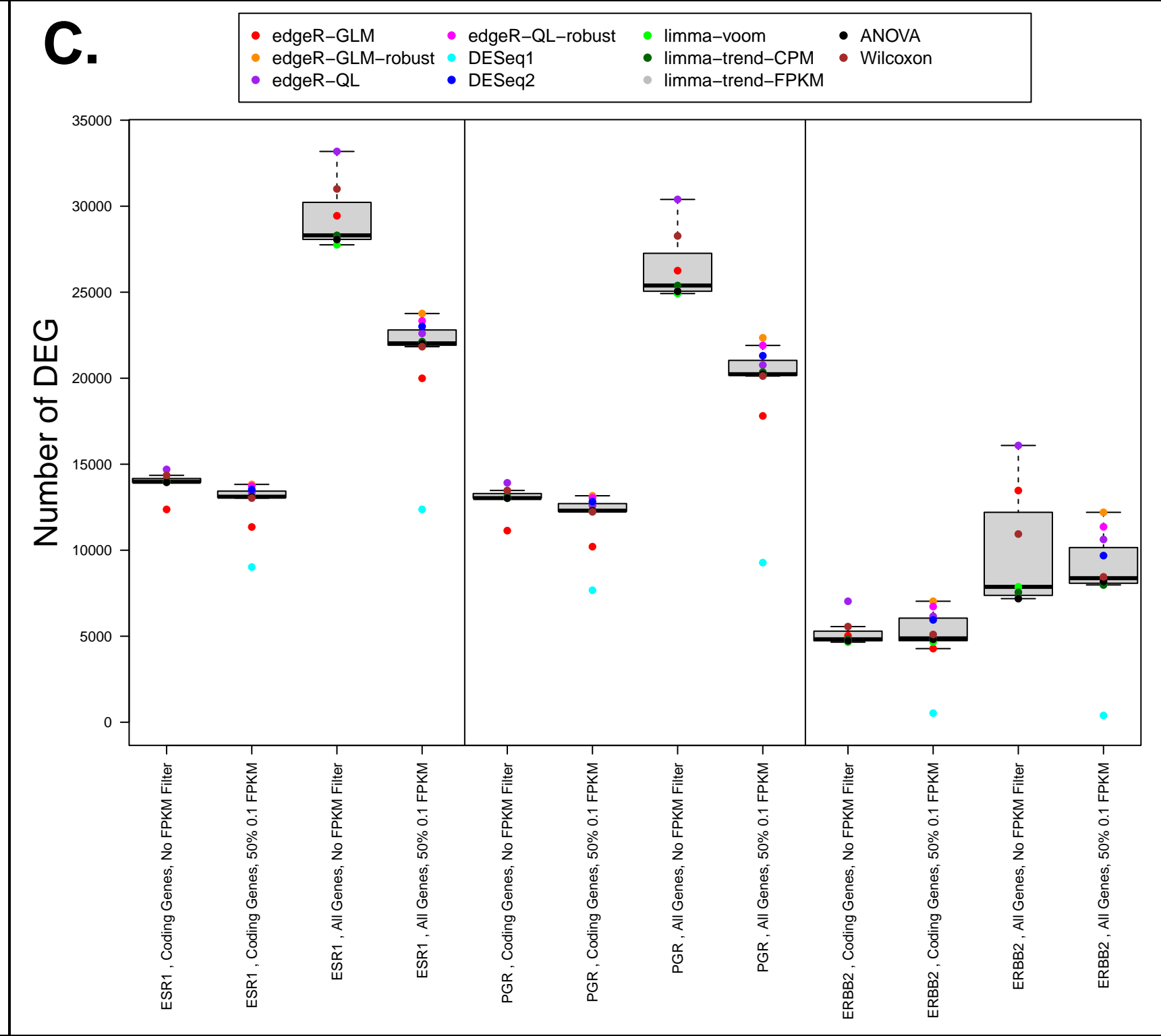

Supplement: Supplement 13 [file media-13.pdf]
